# Effect of Dry Roasting on the Physicochemical, Nutritional, and Techno-Functional Properties of Tri-Color Quinoa Flours

**DOI:** 10.3390/foods14183237

**Published:** 2025-09-18

**Authors:** Yvette Mukunzi, Alberta N. A. Aryee

**Affiliations:** Department of Human Ecology, College Agriculture, Science and Technology, Delaware State University, 1200 N DuPont Highway, Dover, DE 19901, USA

**Keywords:** quinoa, dry roasting, nutritional quality, antinutritional factors, techno-functional properties

## Abstract

Quinoa (*Chenopodium quinoa*), a gluten-free pseudocereal of increasing interest in food applications, remain underutilized due to limited knowledge of its nutritional and techno-functional properties, particularly following processing. This study investigated the impact of roasting on these properties of tri-color quinoa. Roasting resulted in non-significant increases in the content of protein, lipid, and starch fractions, while carbohydrate and energy contents increased significantly (*p* < 0.05) by 3.74 and 3.30%, respectively, compared to native tri-color quinoa flour (NTQF). Notably, total dietary fiber, phytic acid, and oxalate contents were decreased by 13.11, 36.05, and 28.78%, respectively, contributing to improvements in in vitro protein digestibility and in vitro protein digestibility-corrected amino acid score in roasted tri-color quinoa flour (RTQF). Although lysine remained the limiting amino acid, its content increased in RTQF. Techno-functional properties were also affected by roasting; water and oil absorption capacities increased by 24.26 and 2.76% (*p* < 0.05), while emulsifying, foaming, and swelling capacities declined by 47.58, 34.96, and 17.74%, respectively (*p* < 0.05). RTQF exhibited consistently lower protein solubility across all pH tested, and higher a least gelation concentration, likely due to protein denaturation. Color analysis showed darker (L*), redder (a*), and more yellow (b*) hues in RTQF, with minor but perceptible color difference (ΔE = 1.26) relative to NTQF. Scanning electron microscopy revealed greater starch disruption, increased porosity and fragmentation in RTQF than NTQF. FTIR confirmed structural alterations, with the spectrum of RTQF showing less intense bands and higher transmittance compared to NTQF, associated thermal modification of carbohydrate, moisture content and other components. These findings suggest that dry roasting can be used to modify the nutritional and techno-functional properties of tri-color quinoa, offering expanded opportunities for tailored food applications.

## 1. Introduction

Gluten-free ancient grains such as quinoa, amaranth, buckwheat, sorghum, teff, and millet are increasingly popular alternative ingredient sources for various applications [1]. Among these, quinoa (*Chenopodium quinoa Willd*), native to the Andes, stands out for its exceptional nutritional profile, making it a valuable addition in food formulations. Among the many different varieties of quinoa are those with distinctively colored seed coats such as red, white, and black. Tri-color quinoa is a blend of these three color types, offering more diverse nutritional, functional, and flavor profiles compared to single-color quinoa. Quinoa is rich in protein (12–16.5%), lipid (5–9%), carbohydrate (60–74.7%), and essential fatty acids such as linoleic acid [2,3]. It contains a balance of all the essential amino acids (EAA), making it superior to many other grains [1,4], an ideal complement to legumes [5]. In addition to its rich nutritional profile, quinoa exhibits desirable techno-functional properties such as high water and oil absorption capacity, good emulsifying and foaming ability, and gelation behavior, which contribute to its versatility in food formulations [2,6]. However, it contains antinutritional factors (ANFs) such as saponins, phytates, oxalates, tannins, phytic acid, and trypsin inhibitor, which can form insoluble complexes with nutrients, impairing their utilization and absorption [7,8].

Various processing techniques such as soaking, thermal treatment, germination, and fermentation have been applied to reduce the content of ANFs, improve nutritional quality, and techno-functional properties of various legumes, pulses, and grains [7,8,9]. Apart from these, roasting has also been shown to modify color and enhance flavor profile mainly due to the Maillard reaction [2,10]. Additionally, studies have shown that roasted composite flours of sorghum, millet, chickpea, and green gram exhibit higher net protein consumption compared to their boiled or baked counterparts [1,11]. Although various processing methods, including roasting and fermentation, have been studied for single-color quinoa varieties (red, white, or black), limited information exists on tri-color quinoa. This blend exhibits unique compositional and structural interactions that influence its response to roasting, resulting in distinct nutritional and functional outcomes. To address this gap, the present study investigates the effects of dry roasting on the nutritional quality, ANFs content, and techno-functional properties of tri-color quinoa flour. The study aims to provide a comprehensive characterization of these properties to support its application in gluten-free, plant-based, and health-oriented food formulations

## 2. Materials and Methods

### 2.1. Materials

Unpolished tri-color quinoa, a blend of red, white, and black quinoa seeds, were provided by Ardent Mills LLC (Denver, CO, USA). Sunflower oil was purchased from Walmart (Dover, CO, USA). Assay kits of phytic acid (K-PHYT), resistant starch (Rapid), and total dietary fiber were obtained from Neogen (Lansing, MI, USA). Natural amino acid kit was purchased from Carolina Biological Supply Co. (Burlington, NC, USA), amylo-glucosidase, bovine serum albumin, chymotrypsin, protease, trypsin inhibitor, and a Supelco 37 Component FAME mix were purchased from Sigma-Aldrich (St. Louis, MO, USA). Oxalate assay kit was obtained from Fisher Scientific (Waltham, MA, USA) and DC Protein assay reagents from Bio-Rad Laboratories, Inc. (Hercules, CA, USA).

### 2.2. Methods

#### 2.2.1. Sample Preparation

After sorting the seeds, half of the cleaned whole tri-color quinoa was ground using a KitchenAid coffee blender (Boca Raton, FL, USA) to pass through a 140-mesh sieve. The flours obtained were combined to yield a homogeneous mixture that was labeled native tri-color quinoa flour (NTQF). The other half was roasted using optimized roasting conditions (120 °C and 8 min) on a hot plate, selected based on preliminary studies and existing literature on similar seeds. The roasted seeds were allowed to cool, and ground and mixed as previously described to obtain the roasted tri-color quinoa flour (RTQF).

#### 2.2.2. Physicochemical Characteristics

##### Proximate Analysis, Energy, and Total Dietary Fiber (TDF) Content

Moisture, lipid, and ash content were determined according to AOAC methods 925.10, 920.85, and 923.03, respectively [12]. Protein content was determined following the Dumas method [12] using a CN828 analyzer (LECO Corporation, St. Joseph, MI, USA) (%N × 6.25). Carbohydrate content was calculated by difference, as 100% − [% protein + % lipid + % ash + % moisture]. Energy value was estimated using Atwater factors: 4 kcal/g for protein and carbohydrate, and 9 kcal/g for fat. TDF content was determined according to manufacturer’s protocol. 

##### Resistant and Digestible Starch

The resistant starch (RS) and digestible starch (DS) content were measured using the resistant starch assay kit (rapid). Exactly 100 mg of each sample was weighed into 50 mL falcon tubes and 3.5 mL sodium maleate buffer (pH 6.0) containing 2 mmol/L calcium chloride. The suspension was vortexed, placed in a water bath at 37 °C for 5 min to equilibrate to the desired temperature. Aliquot of 0.5 mL of solution 1 containing 0.4 KU of pancreatic α-amylase and 0.17 KU of amyloglucosidase (AMG) was added to each tube. The tubes were tightly capped and placed in a shaking water bath set at 37 °C with constant shaking (200 strokes per min) for 4 h. Each tube was taken out of the water bath, and then 4.0 mL 95% (*v*/*v*) ethanol was added, followed by vigorous vortexing. The tubes were centrifuged at 1500× *g* for 10 min (Eppendorf centrifuge 5810 R, Hauppauge, NY, USA). The supernatant was decanted and stored for DS measurement. To resuspend the pellets, 2 mL 50% (*v*/*v*) ethanol was added and vortex. The tube was then filled with an additional 6 mL 50% (*v*/*v*) ethanol, sealed, and well mixed by inversion. Tubes were tapped until all liquid was withdrawn from the caps. After removing the caps, the tubes were centrifuged at 1500× *g* for 10 min (Eppendorf centrifuge 5810 R, Hauppauge, NY, USA). The resulting supernatant was then decanted and mixed with the original supernatant. The residue was re-suspended in 8 mL 50% (*v*/*v*) ethanol, centrifuged under the same conditions previously described, and the supernatant was decanted and added to the first two supernatants. To remove excess liquid and prevent pellet dislodgement, the tubes with the residue were placed on absorbent paper and inverted. Aliquot of 2 mL cold 1.7 mol/L NaOH was added to each tube, and the pellets were mixed for ≈20 min in an ice/water bath. Each tube was filled with 8 mL sodium acetate buffer (1.0 M, pH 3.8), which included 5 mmol/L calcium chloride. AMG (0.1 mL, 3300 U/mL) was directly added; the tubes were thoroughly mixed before being placed in a water bath at 50 °C and incubated for 30 min with constant mixing. The samples with a 10% RS content were completely transferred to a 100 mL volumetric flask using a water wash bottle. The volume was adjusted to 100 mL with Milli-Q water and thoroughly stirred. Aliquots of each solution were centrifuged at 1500× *g* for 5 min (Eppendorf centrifuge 5810 R, Hauppauge, NY, USA). Duplicate aliquots (0.1 mL) of all solutions were transferred to the glass test tubes, and 3.0 mL glucose determination reagent (GOPOD reagent) was added while mixing. The tubes were then incubated at 50 °C for 20 min. A reagent blank solution was created by combining 0.1 mL 100 mmol/L acetic acid (pH 4.5) with 3.0 mL GOPOD reagent. Glucose standards (in quadruplicate) were made by combining 0.1 mL glucose solution (1 mg/mL) and 3.0 mL GOPOD reagent and incubated at 50 °C for 20 min. The absorbance of each solution was measured at 510 nm against a reagent blank, and the RS content was calculated. The combined supernatants were adjusted to 100 mL with 100 mmol/L sodium acetate buffer (pH 4.5), and a 2.0 mL aliquot was centrifuged at 1500× *g* for 5 min (Eppendorf centrifuge 5810 R, Hauppauge, NY, USA). To determine DS content, duplicate aliquots (0.1 mL) were transferred to glass tubes, 0.1 mL AMG (100 U/mL) in 100 mmol/L sodium acetate buffer (pH 4.5) was added, and the tubes were incubated at 50 °C for 30 min. About 3.0 mL of GOPOD reagent was then added to the tubes, and they were incubated at 50 °C for 20 min. A reagent blank solution was generated by combining 0.2 mL of 100 mmol/L acetic acid (pH 4.5) with 3.0 mL GOPOD reagent. Glucose standards (in quadruplicate) were prepared by combining 0.1 mL glucose solution (1 mg/mL) with 0.1 mL 100 mmol/L sodium acetate buffer (pH 4.5) and 3.0 mL GOPOD reagent, then incubating at 50 °C for 20 min with the sample solution. The absorbance of each solution was measured at 510 nm against a reagent blank, and the DS content was calculated.

##### Fatty Acid (FA) Composition

FA composition was determined using the method described by Vujić et al. [13]. To 0.5 g of flour was added 5 mL n-hexane, and the mixture was vortexed for 2 min. The supernatant was collected after centrifuging at 645× *g* for 5 min (Thermo Scientific Heraeus^®^ Megafuge^®^ 8, Waltham, MA, USA). An aliquot of 3 mL of the supernatant was left to evaporate at room temperature, and 10 µL of the remaining oily residue was collected and reconstituted in 400 µL of methanol to which 10 µL (10 mg/mL) of heptadecanoic acid internal standard was added. The sample was transesterified by adding 100 µL trimethylsulfonium hydroxide (0.2 mol/L in methanol). The FA methyl esters (FAME) formed were analyzed using a gas chromatography (GC) system (GC-2010 Plus, Shimadzu, Kyoto, Japan) equipped with a flame ionization detector (FID). Separation was carried out using a Zebron ZB-FAME column (30 m × 0.25 mm × 0.20 µm, Phenomenex, Torrance, CA, USA). The temperature program involved an initial increase from 100 to 140 °C at a rate of 3 °C/min, followed by a further rise from 140 to 260 °C at 30 °C/min, with a hold time of 2 min. The injector temperature was maintained at 240 °C, with helium as the carrier gas at a constant flow rate of 1.2 mL/min. Injection was performed in split mode (60:1) at 1 µL of the sample solution. The FAME Component mix was run in tandem. FAMEs were identified by comparing their relative and absolute retention times to those of the standard, and FA composition was reported as relative percent of total peak area.

##### Mineral Content

The flour samples were analyzed using inductively coupled plasma optical emission spectrometry (ICP-OES) after being microwave digested with HNO_3_, following the method described by Isaac & Johnson [14]. The analysis was conducted by the Service Testing and Research (STAR) Laboratory at Ohio State University. Duplicate analyses were conducted on a suite of 13 minerals including aluminum (Al), boron (B), calcium (Ca), copper (Cu), iron (Fe), potassium (K), magnesium (Mg), manganese (Mn), molybdenum (Mo), sodium (Na), phosphorus (P), sulfur (S), and zinc (Zn), and concentration was reported as mg/kg.

##### Anti-Nutritional Factors (ANFs)

Phytic Acid Content

Phytic acid content was measured using the phytic acid kit (K-PHYT) in triplicate according to the manufacturer’s protocol. Briefly, 10 mL 0.66 M HCL was added to 0.5 g of each flour sample and mixed thoroughly on an Orbi-Shaker^TM^ XL (Benchmark Scientific, Edison, NJ, USA) at 95 rpm for 3 h at room temperature. Aliquot of 1 mL of the mixture was centrifuged at 15,000× *g* for 10 min (Eppendorf centrifuge 5424 R, Enfield, CT, USA). Then, 0.5 mL of the supernatant was transferred to another tube and neutralized by adding 0.5 mL 0.75 M NaOH. To 0.05 mL neutralized extract, 0.62 mL Milli-Q water, 0.2 mL sodium acetate buffer (pH 5.5), and 0.02 mL phytase enzyme (provided in the kit) were added. The mixture was vortexed and incubated in a water bath at 40 °C for 10 min. Then 0.02 mL each of Milli-Q water, glycine buffer (from the kit), and alkaline phosphatase (from the kit) were added, vortexed, and incubated again in a water bath at 40 °C. The reaction was stopped after 15 min by adding 0.30 mL 50% (*w*/*v*) trichloroacetic acid. The samples were then centrifuged at 15,000× *g* for 10 min (Thermo Scientific Heraeus^®^ Megafuge^®^ 8, Waltham, MA, USA). Then, 150 µL of the supernatant from each sample and a 150 µL phosphorus standard were mixed with 150 µL of color reagent (a 1:5 mixture of 5% (*w*/*v*) ammonium molybdate and 10% (*w*/*v*) ascorbic acid in 1 M sulfuric acid) and incubated at 40 °C for 1 h. The absorbance of the resulting mixture was measured at 655 nm using BioTek Synergy HTX multi-mode reader (Agilent, Santa Clara, CA, USA). Phytic acid content was expressed as mg/g.

Saponin Content

Saponin content was determined using the method described by Bhinder et al. [15]. Briefly, 0.5 g of each flour sample was mixed with 10 mL 80% methanol (*v*/*v*) in water and vortexed. The mixture was agitated for 16 h and then centrifuged for 10 min at 4500× *g* (Eppendorf centrifuge 5810 R, Hauppauge, NY, USA). The supernatant was transferred into another tube, to which an additional 5 mL 80% (*v*/*v*) methanol was added, followed by centrifugation at 4500× *g* for 10 min (Eppendorf centrifuge 5810 R, Hauppauge, NY, USA). This step was repeated twice, and supernatants were pooled. Aliquot of 200 μL of the pooled extract was mixed with 50 μL 80% (*v*/*v*) methanol. The tubes were then placed in an ice bath, and 25 μL vanillin reagent (80 mg/mL in methanol) and 250 μL 72% sulfuric acid were added. The tubes were vortexed and incubated in a water bath at 60 °C for 10 min. The absorbance of the samples was read at λ = 520 nm against Milli-Q water as blank using a BioTek Synergy HTX multi-mode reader (Agilent, Santa Clara, CA, USA). A calibration curve for diosgenin (10–120 mg/mL) was developed to extrapolate saponin content (mg/g).

Tannin Content

Tannin content was determined as described by Bhinder et al. [15] using the vanillin-hydrochloric acid method. Samples of 200 mg flour were extracted in 10 mL of absolute methanol for 20 min. The mixture was centrifuged at 3000× *g* for 10 min (Thermo Scientific Heraeus^®^ Megafuge^®^ 8, Waltham, MA, USA), and the supernatant was collected for the analysis. A working solution of vanillin reagent was prepared using a mixture of 1% vanillin in methanol and 8% HCl in methanol in a 1:1 ratio (*v*/*v*). Following this, the working vanillin and catechin solutions were incubated in a water bath set at 30 °C for 30 min. Then, 50 µL of flour extract was pipetted into a 96-well plate, with one well each for control and the sample. A 250 µL vanillin reagent was added to the sample wells, while 150 µL 4% (*v*/*v*) HCl was added to the control wells after 1 min. The plates were incubated for 20 min and then read using a BioTek Synergy HTX multi-mode reader (Agilent, Santa Clara, CA, USA), with a 1 min interval between reading sample and control to maintain consistency. Sample controls were used to prevent false positives, and methanol served as the blank. Tannin content was calculated from a catechin standard curve (0.06–0.30 mg/mL) and expressed as mg catechin equivalents (CE) per gram of sample.

Oxalate Content

Oxalate content was measured using an oxalate assay kit, following manufacturer’s protocol. Briefly, 15 mg of each sample was homogenized in 150 µL assay buffer from the kit, incubated on ice for 10 min, and then centrifuged at 10,000× *g* for 5 min (Eppendorf centrifuge 5424 R, Enfield, CT, USA). In a 96-well plate, 50 µL oxalate standard with concentrations of 2, 4, 6, 8, and 10 mmol/L were added to the standard wells. For the sample wells, 20 µL of each sample was pipetted, and the volume was adjusted to 50 µL with the assay buffer. Then, 2 µL oxalate converter was added to the samples and standard in the wells. After mixing thoroughly, the plate was incubated at 37 °C for 1 h to allow for conversion. Following this, 50 µL reaction mix was added to each well and incubated for another 60 min at 37 °C, protected from light. Absorbance was read at 450 nm, and oxalate content (mg/g) was extrapolated from the standard curve.

##### Trypsin Inhibitor Activity (TIA)

TIA was assessed following the method by Adeleye et al. [16] using N-benzoyl-DL-arginine-ρ-nitroanilide (BAPNA) as the substrate. In brief, 0.08 g defatted flour was mixed with 3 mL 0.01 mol/L NaOH and incubated in a PS 30A ultrasonic water bath (Eujgoov, China) for 3 h at 23 °C, maintaining a pH 9.5–9.8. Aliquot of 200 µL of the sample and 200 µL 40 µg/mL trypsin was pre-incubated at 37 °C for 3 min. The reaction was initiated by adding 500 µL 0.4 mg/mL BAPNA, pre-warmed to 37 °C. After 10 min of incubation at 37 °C, the reaction was terminated by adding 100 µL of 30% (*v*/*v*) acetic acid, followed by centrifugation at 2778× *g* for 10 min (Eppendorf centrifuge 5810 R, Hauppauge, NY, USA). TIA was quantified by measuring the release of ρ-nitroaniline at 410 nm using a BioTek Synergy HTX multi-mode reader (Agilent, Santa Clara, CA, USA). One unit of trypsin inhibitor was defined as a 0.01 decrease in absorbance at 410 nm under assay conditions compared to the control without inhibitor.

##### Water Activity and Color Parameters

Water activity (a_w_) and color parameters were measured with an Aqualab meter (Decagon devices, Pullman, WA, USA) at 25 °C and portable CR-10 plus colorimeter (Konica Minolta, Tokyo, Japan), respectively. The color analysis measured the luminosity (L*), redness (a*), and yellowness (b*) and color difference (ΔE) was calculated as

(1) $$\Delta E=\sqrt{{\left(L_{1}^{*}-L_{2}^{*}\right)}^{2}+{\left(a_{1}^{*}-a_{2}^{*}\right)}^{2}+{\left(b_{1}^{*}-b_{2}^{*}\right)}^{2}}$$

where 1 and 2 refer to the colorimetric value of native and roasted flours, respectively.

##### Thermal Properties

The thermal characteristics of the flours was evaluated using a DSC-400 differential scanning calorimeter (PerkinElmer, Waltham, MA, USA). The DSC was calibrated with an indium standard. Flour samples (10 mg) were weighed in aluminum pans and hermetically sealed. The reference material used was an empty aluminum pan. The pans were heated from 25 to 200 °C at a rate of 5 °C/min, and the nitrogen flow rate of 50 mL/min was maintained throughout the experiment. The thermograms were then analyzed using Pyris v11 Data Analysis software (Perkin Elmer, Waltham, MA, USA). The onset temperature (To), peak temperature (Tp), end temperature (Te), and the enthalpy of gelatinization (ΔH) of the samples were evaluated.

##### Secondary Structure Analysis

Fourier transform infrared (FTIR) spectroscopy was employed for secondary structure analysis. FTIR spectra were recorded between 450 and 4000 cm^−1^ with a resolution of 4 cm^−1^ using a Cary 630 FTIR spectrometer (Agilent, Santa Clara, CA, USA) with diamond ATR module. Air was used as the background for the scans. Samples were carefully placed on the diamond crystal of the base plate, and the pressure arm was lowered to ensure proper contact with the crystal surface. Complete FTIR spectra were obtained and compared to an open beam background spectrum. Both flours were analyzed three times, and the average spectrum was recorded.

##### Microstructure and Elemental Analysis

Scanning electron microscopy (SEM) and energy-dispersive X-ray spectroscopy (EDS) were utilized to examine the microstructure and elemental composition of the flours. A high-resolution environmental SEM (Quanta 250 FEG, FEI Co., OR, USA) operating at 30 kV was employed, equipped with an energy-dispersive X-ray spectrometer (XFlash 5010; Bruker AXS Microanalysis, Berlin, Germany). The flours were mounted onto a specimen holder and examined three times at varying magnifications, ranging from 500× to 1000×. This analytical approach produced a chromatogram that illustrated the elemental composition in terms of weight percentage, with each element displaying a unique peak pattern. The analysis accounted for all detectable elements, except for hydrogen, helium, and lithium, which did not exhibit excited X-ray peaks. Elements present in concentrations greater than 0.1% by weight were classified as major components, while those with concentrations between 0.001% and 0.01% were considered minor constituents, with the minimum detection threshold set at 0.001%.

#### 2.2.3. Protein Quality

Amino acid composition was determined using the method of Li et al. [17] with slight modification. Briefly, 0.5 g of sample flour was extracted with 10 mL 75% (*v*/*v*) ethanol, homogenized for 10 min on ice, and centrifuged at 250× *g* for 15 min at 4 °C (Eppendorf centrifuge 5810 R, Hauppauge, NY, USA). The resulting supernatant was collected and stored at 4 °C until analysis. Tryptophan and cysteine standards were prepared as 5 mg/mL solution in methanol. For derivatization, 180 μL of the sample extract, standard mixture, and tryptophan and cysteine standards were transferred into separate reaction tubes. To each tube, 100 μL 2,4-dinitrofluorobenzene, 100 μL sodium bicarbonate, and 20 μL Milli-Q water were added. The mixtures were incubated at 60 °C for 1 h in the dark to complete derivatization. After incubation, 400 μL potassium dihydrogen phosphate buffer was added to each tube to stabilize the derivatives. Amino acid analysis was conducted using an LC-20AB HPLC system equipped with a DGU-20A3R degasser, SIL-20AC autosampler, CTO-20AC column oven, and SPD-M20A diode array detector (Shimadzu Co., Kyoto, Japan). Instrument control and data acquisition were managed by LC solution software, version 5.124 SPI (Shimadzu Co., Kyoto, Japan). HPLC conditions included a ProntoSIL C18 column (120 Å, 5 μm, 4.6 × 250 mm; Agilent Technologies Inc., Santa Clara, CA, USA); 20 μL injection volume; oven temperature at 35 °C; and a flow rate of 0.6 mL/min. The eluate was monitored at 360 nm. Amino acid contents were quantified based on peak areas using calibration curves constructed from amino acid standards (0.2–4.1 µg/mL). The mobile phases consisted of solvent A (5 mmol/L sodium acetate buffer, pH 5.7, with tetrahydrofuran in a 95:5, *v*/*v* ratio) and solvent B (80% methanol). The gradient elution program began with solvent A at 90% and solvent B at 10%, gradually adjusting over the first 20 min to A (80%) and B (20%). From 20 to 25 min, solvent A was reduced to 50% and solvent B increased to 50%. Between 25 and 34 min, solvent A was further reduced to 45%, with solvent B, increased to 55%. A sharp reduction in solvent A to 0% and increase in solvent B to 100% occurred from 34 to 50 min. The system was re-equilibrated to the initial conditions (90% A, 10% B) from 50 to 55 min, and held constant until 60 min.

The amino acid score (AA score) was calculated as the ratio of the amino acid content (mg) in 1 g of the test protein to that of the reference protein. The amino acid requirements for various ages groups including; infants (0.5 y), preschool children (1–2 y), adolescents (11–14 y), and adults (>18 y) recommended by the Food and Agriculture Organization (FAO) was used [18]. The limiting amino acid is the EAA present in the lowest quantity relative to the reference protein.

In vitro protein digestibility (IVPD) was determined using the method described by Hsu et al. [19]. Flour containing the equivalent of 62.5 mg protein was weighed and hydrated with 10 mL Milli-Q water, then equilibrated at 37 °C for 1 h. The pH was adjusted to 8.0 using 0.1 mol/L NaOH or HCl. An aliquot of 1 mL multienzyme cocktail was prepared by combining 16 mg trypsin (13,000–20,000 BAEE units/mg protein), 31 mg chymotrypsin (40 units/mg protein), and 13 mg protease (50–100 units/g solids) in 10 mL Milli-Q water at 37 °C (pH 8.0) and then added to each slurry. The pH was recorded at 0 min and 10 min of incubation at 37 °C. IVPD was calculated as follows:

(2) $$\mathrm{IVPD}\left(\%\right)=65.66+18.10\;\Delta \;pH_{10\min}\;$$

where ΔpH_10min_ is the change in pH from 0 to 10 min.

In vitro protein digestibility corrected amino acid score (IVPDCAAS) was calculated as a product of the AA score and IVPD values.

The essential amino acid index (EAAI) represents the ratio of all EAA of the test protein to the amounts of those same amino acids of a human requirement (reference protein) for infants (0.5 y), preschool children (1–2 y), adolescents (11–14 y), and adults (>18 y) [18]. EAAI was calculated as the geometric mean of the ratios of each EAA in the test protein to its corresponding amount in the reference protein. The predicted biological value (p-BV) is another index that reflects the human body’s efficiency in absorbing proteins from diet [20]. p-BV were calculated as follows:

(3) p−BV= 1.09 × EAAI−11.70

#### 2.2.4. Techno-Functional Properties

##### Protein Solubility

Protein solubility of the flours at pH 2–12 was determined using the method described by Stone et al. [9]. To a 0.3 g sample was added 30 mL Milli-Q water and vortexed for 1 min. The pH was adjusted to a range of 2 to 12 using 0.5 N HCl or NaOH, and the mixture was stirred for 1 h at room temperature using a magnetic stirrer. The mixture was then examined, adjusted up to the testing pH, and centrifuged at 800× *g* for 10 min (Eppendorf centrifuge 5810 R, Hauppauge, NY, USA). The supernatant was transferred into another tube and the volume made up to 35 mL with Milli-Q water. The mixture was centrifuged again for 10 min at 800× *g*. The supernatant was analyzed for protein content using the Bradford assay. Protein solubility was expressed as a percentage, calculated by the proportion of protein present in the supernatant relative to the total protein content of the sample.

##### Water Absorption and Oil Absorption Capacity (WAC and OAC)

WAC and OAC were determined as described by Stone et al. [9]. Portions of 1 g of each sample was weighed into a tube and 10 mL of Milli-Q water/oil was added. Each of the sample tubes was vortexed for 1 min and was incubated at room temperature for 30 min and then centrifuged for 30 min at 2778× *g* (Eppendorf centrifuge 5810 R, Hauppauge, NY, USA). The samples were then drained for 15 min and reweighed. WAC and OAC were expressed as g/g.

##### Emulsifying Capacity and Stability (EC and ES)

EC and ES were determined using the method described by Stone et al. [9]. To prepare the emulsion, 0.5 g of the sample was added to a graduated centrifuge tube, and 10 mL of Milli-Q water, and 10 mL of soybean oil was added and vortexed. The mixture was then centrifuged at 694× *g* for 5 min (Eppendorf centrifuge 5810 R, Hauppauge, NY, USA). For ES, the emulsion in the graduated centrifuge tube was heated at 80 °C for 30 min in a water bath, then cooled for 15 min, followed by centrifugation at 694× *g* for 15 min (Eppendorf centrifuge 5810 R, Hauppauge, NY, USA). EC (%) was calculated by diving the volume of emulsified layer by the total content of the tube (×100), while the ES also expressed as a percentage was calculated as the volume of emulsified layer after heating relative to the volume of the emulsified layer before heating.

##### Foaming Capacity and Stability (FC and FS)

FC and FS were determined as described by Sohaimy et al. [21]. Briefly 10 mL 3% (*w*/*v*) flour samples dispersed in Milli-Q water were homogenized using a Pulse 150 Ultrasonic Homogenizer (Benchmark Scientific, NJ, USA) at a high setting for 3 min. The mixture was then immediately transferred to a graduated cylinder, and the homogenizer cup was rinsed with 10 mL of Milli-Q water and added to the cylinder. The volume was recorded before and after whipping. FS was measured by recording the volume changes in the graduated cylinder after standing for 20, 40, 60, and 120 min. FC (%) was calculated as the difference in foam volume before and after homogenization relative to the volume before homogenization, while FS (%) was calculated as the foam volume at a specified time divided by the initial foam volume.

##### Swelling Capacity (SC)

SC was determined using the method described by Sharma et al. [7], with some modifications. In brief, 10 mL Milli-Q water was added to 1 g of flour and then vortexed. The suspension was left to rest for 20 h at room temperature (23 °C). The bed volume (mL) was then measured, and the SC was calculated as the final volume occupied by the hydrated flour.

##### Least Gelation Concentration (LGC)

LGC was determined using the test tube inversion method as described by Badia-Olmos et al. [22]. Sample solutions were prepared in 10 mL tubes with suspensions of 2, 4, 6, 8, 10, 12, 14, 16, 18, and 20% (*w*/*v*) of the flours. The samples were heated for 30 min at 95 °C in a water bath, then cooled to room temperature for 1 h, and then left overnight at 4 °C. The tubes were then inverted to check for gelling. The LGC is the concentration above which the samples did not drop or slip when the test tubes were inverted.

### 2.3. Statistical Analysis

The data obtained were analyzed using Minitab (Version 7). Analysis of variance and significant differences were analyzed by the Fisher LSD method followed by Tukey’s post hoc tests (*p <* 0.05) in the same procedure to determine significant differences between NTQF and RTQF. Results were expressed as mean values from three replicates (unless otherwise stated), accompanied by the standard deviation. Pearson correlation analysis was performed using the “correlation” package (v0.8.6) in R to assess significant relationships among the physicochemical, nutritional, and techno-functional properties of the flours. The results were visualized with the “corrplot” package (v0.95).

## 3. Results and Discussion

### 3.1. Physicochemical Characteristics

#### 3.1.1. Proximate Composition and Energy Content

The proximate composition of the flours is presented in Table 1. Apart from moisture and carbohydrate content, there were no significant differences between the NTQF and RTQF. The moisture content of NTQF was consistent with the 7.93 and 7.99% reported for raw white quinoa by Beniwal et al. [1] and Norouzian et al. [10], respectively. However, it was notably lower than the values reported for red, black, and yellow quinoa flours by Manzanilla-Valdez et al. [23] (10.90, 11.53, and 12.04%, respectively). Similarly, Abdelmegiud et al. [24] reported differences in moisture content among quinoa, buckwheat, millet, and chickpeas (10.34, 10.72, 11.16, and 11.43%, respectively). Roasting significantly reduced the moisture content by 37.80% compared to NTQF, due to moisture loss [4]. Similar reductions in moisture content following roasting have been reported in quinoa flour by Castro-Alba et al. [4], Beniwal et al. [1], and Norouzian et al. [10], with decreases of 67.09, 56.38, and 21.15%, respectively. The lower moisture content can reduce microbial growth and extend the shelf life of the flour [1].

The ash content of NTQF was lower than the values reported by Beniwal et al. [1] (3.46%) and Marie et al. [8] (2.78%), and notably higher than that reported by Norouzian et al. [10] (0.99%), likely attributed to varietal differences, milling process and refinement. It was also lower than the ash content reported in red (2.58%) and black (2.57%) quinoa flours, but higher than that of yellow quinoa flour (2.32%), as noted by Manzanilla-Valdez et al. [23]. However, RTQF showed a 3.67% higher ash content compared to NTQF. This may be due to the concentration of minerals due to process-induced loss and changes to other components [4]. Marie et al. [8], and Norouzian et al. [10], respectively, reported 7.02 and 15.38% increases in ash content in quinoa with roasting. Beniwal et al. [1] reported a significant reduction of 66.42% in ash content in roasted quinoa flour. This variation may be attributed to varietal differences, roasting temperature, duration, or leaching during processing [23].

Furthermore, the lipid content of NTQF was notably lower than values reported by Beniwal et al. [1] (8.1%), Marie et al. [8] (7.41%), and slightly lower than that reported by Norouzian et al. [10] (6.18%). Conversely, it was higher than the lipid contents reported for yellow (3.89%), red (4.86%), and black (5.15%) quinoa flours by Manzanilla-Valdez et al. [23]. These variations are likely due to differences in variety, and lipid extraction methods employed. Roasting had no significant effect on lipid content, although a nominal increase of 3.76% was observed, compared to NTQF, likely due to enhanced lipid extractability and quantification following moisture loss during roasting, which facilitates solvent access to lipid-rich structures [4,23]. The lipid content in RTQF was lower than the 6.06, 6.31, and 5.96% reported by Beniwal et al. [1], Marie et al. [8], and Norouzian et al. [10], respectively. The difference is likely due to the variations in variety, roasting conditions, and extraction methods [23].

Additionally, the protein content of NTQF was notably higher than the value reported by Norouzian et al. [10] (3.31%), and slightly higher than that by Marie et al. [8] (13.98%) and Beniwal et al. [1] (14.5%). However, it was notably lower than the values reported for yellow, red, and black quinoa flours by Manzanilla-Valdez et al. [23] (19.34, 20.25, and 20.90%, respectively). This may be attributed to differences in variety, cultivation, and analytical methods used. No statistically significant effect of roasting was observed for protein content, though it was 0.87% higher in the RTQF compared to NTQF, likely attributed to moisture reduction, which concentrates the solid components of the flour [23]. Marie et al. [8] reported a 2.71% increase in protein content in roasted quinoa flour compared to the raw counterpart. The protein content in RTQF was lower than the 13.20 and 14.37% reported by Beniwal et al. [1] and Norouzian et al. [10], respectively, in roasted quinoa flour, likely due to differences in roasting conditions, such as temperature, duration, and method [10].

Moreover, the carbohydrate content of NTQF was comparable to the 70.85% reported by Marie et al. [8], but lower than the 81.50% reported by Norouzian et al. [10] and higher than the values reported for black, yellow, and red quinoa flours by Manzanilla-Valdez et al. [23] (60.12, 66.90, and 68.10%, respectively). Roasting significantly enhanced carbohydrate content by 3.74%, consistent with trends observed by Norouzian et al. [10] (2.49%), but notably higher than the 0.93% recorded by Marie et al. [8]. This thermal dehydration concentrates macronutrients, including carbohydrates, on a dry weight basis [4].

Furthermore, the energy value of NTQF was slightly lower than reported by Norouzian et al. [10] (394.98 kcal/100 g), and notably lower than the 465.51 kcal/100 g recorded by Marie et al. [8], likely attributed to differences in macronutrient composition. Energy value of RTQF was 3.30% higher than that of NTQF, and the difference was statistically significant. The reduction in moisture enhances caloric density by concentrating energy-yielding nutrients per unit weight, with carbohydrates serving as a primary energy source [4,25]. Similarly, Norouzian et al. [10] and Marie et al. [8] reported increases of 1.24 and 0.25%, respectively, in the energy value of roasted compared to the unroasted quinoa flour.

#### 3.1.2. TDF and Starch Content

The TDF content in NTQF was notably higher than the value reported by Badia-Olmos et al. [11] (8.7%), likely due to differences in processing methods such as milling, which can influence the retention of fiber-rich outer layers of the seed [8]. Moreover, the TDF content was comparable to the range reported by Manzanilla-Valdez et al. [23] (16.15–22.97%) for red, yellow, and black quinoa flour. McCleary et al. [26] also observed significant in TDF content of wheat, corn, and rice, with values of 10.7, 7.3, and 2.8%, respectively. Roasting significantly reduced TDF content by 13.11%, possibly due to heat-induced breakdown of structural polysaccharides such as cellulose and pectin [4]. Similarly, Marie et al. [8] reported a 3.22% decrease in TDF content in roasted quinoa flour. Comparable reductions have been observed in other roasted grains, with Miraji et al. [27] reporting a 7.14% decrease in roasted black chickpea and Ejoh & Onyeulo [28], a 12.5% reduction in TDF content in roasted maize.

Digestible starch content of NTQF was notably lower than the range reported by Manzanilla-Valdez et al. [23] (44.28–45.90%) for red, yellow, and black quinoa. Similarly, the resistant starch content was also notably lower than the reported range of 8.90–10.29% by the same authors. Furthermore, the total starch content of NTQF was lower than the values reported by Diaz-Valencia et al. [29] (47.22–59.72%) and Manzanilla-Valdez et al. [23] (53.41–54.8%), likely due to differences in composition and processing, which can influence starch content and hydrolysis. Roasting led to a slight, non-significant increase of 3.95% in total starch content. Similarly, digestible starch and resistant starch content increased by 2.24 and 10.14%, respectively, with roasting. In comparison, Dong et al. [30] reported higher values in digestible starch (18.35%) and resistant starch (5.32%) contents in heat-treated quinoa. These variations may be due to thermal modification of starch granules under low-moisture conditions, which can cause partial structural disruption without complete gelatinization. Additionally, the cooling phase after roasting likely facilitated starch retrogradation, contributing to higher resistant starch content [23]. Similarly, Kheto et al. [2] reported a 0.14% increase in total starch content in roasted quinoa flour, while Dong et al. [30] reported a slight increase of 0.20% in the total starch content of quinoa after thermal processing. Stone et al. [9] reported 9.14, 3.76, and 19.28% decreases in resistant starch and 1.25, 1.82, and 2.77% increases in total starch content in roasted green lentil, navy bean, and chickpea, respectively.

#### 3.1.3. Fatty Acid Composition

Oleic acid was the most abundant fatty acid in NTQF, followed by linoleic, palmitic, linolenic, and stearic acids. The contents of myristic, palmitic, linoleic, linolenic, erucic, and arachidonic acids in NTQF were lower than the values reported by Ahmed et al. [25] (0.17, 8.91, 59.52, 5.14, 1.07, and 1.19%, respectively) and Babiker et al. [3] (0.20, 16.42, 37.31, 3.32, 1.50, and 0.18%, respectively). Conversely, stearic and oleic acids in NTQF were considerably higher than the values reported by Ahmed et al. [25] (0.75 and 22.41%) and Babiker et al. [3] (1.40 and 37.80%), respectively. These differences may be attributed to varietal differences, and seed maturity, which influence fatty acid biosynthesis. With the exception of linoleic, arachidic, and docosanoic acids, roasting significantly affected the fatty acid composition of the flours (Table 2). Saturated fatty acids (SFA), particularly myristic, palmitic, and stearic acids, showed notable increases of 40.0, 21.84, and 30.32%, respectively, in RTQF compared to NTQF, likely due to the thermal breakdown of triacylglycerols and phospholipids, which facilitated the release of esterified SFA particularly myristic, palmitic, and stearic acids, resulting in higher extractability and consequently elevated contents in the roasted flour [25]. Similarly, oleic acid, a monounsaturated fatty acid (MUFA), significantly increased by 34.43% after roasting. The increase in oleic acid content may enhance the thermal stability, shelf life, and nutritional value of quinoa flour, as it is known to improve lipid oxidative resistance and support cardiovascular health [3]. Conversely, polyunsaturated fatty acids (PUFA) such as linoleic and linolenic acids decreased by 12.87 and 29.54%, respectively, in RTQF, attributable to thermal degradation, as PUFAs are highly sensitive to oxidation during heat processing. Additionally, erucic acid and arachidonic acid, both considered less desirable in high concentrations, were significantly reduced by 52.63 and 60.0%, respectively, suggesting that roasting may improve the quality of quinoa lipids by reducing these fatty acids [31]. Other fatty acids, such as arachidic and docosanoic acids, remained relatively stable after roasting, likely due to their saturated nature and thermal stability, which make them less prone to oxidative degradation [32]. Comparable trends have been observed in previous studies. Ahmed et al. [25] reported increases of 47.06, 2.13, and 2.67% in myristic, palmitic, and stearic acids, respectively, along with a 14.98% decrease in linolenic acid in roasted quinoa flour. Similarly, Babiker et al. [3] reported reductions of 22.58, 21.33, and 27.78% in arachidic, erucic, and arachidonic acids, respectively, after roasting. A similar trend was reported by Babiker et al. [31], where roasting hempseed led to increases in palmitic, stearic, and oleic acids by 3.40, 4.07, and 0.44%, respectively, while arachidic, linoleic, linolenic, and arachidonic acids decreased by 3.57, 1.03, 1.97, and 9.09%, respectively.

#### 3.1.4. Mineral Content

The mineral content of the flours is presented in Table 1. Roasting significantly affected mineral content, with all the minerals except Na showing lower amounts in RTQF compared to NTQF. The mineral content in NTQF was considerably lower than the values reported by Ahmed et al. [25] for Ca (3230.54 mg/kg), Mg (2269.16 mg/kg), Na (1030.57 mg/kg), P (5600.63 mg/kg), Mn (27.95 mg/kg), and Zn (32.29 mg/kg). Conversely, B, Fe, S, and K were notably higher in NTQF than the 9.32, 88.65, 1648.96, and 8842.02 mg/kg, respectively, reported by Ahmed et al. [25]. Manzanilla-Valdez et al. [23] reported higher values for Ca (366.2–478.7 mg/kg), K (4552.1–6322.5 mg/kg), Mg (1451.2–1550.3 mg/kg), and Fe (31.8–41.4 mg/kg) in the three quinoa varieties compared to our study, but a lower value for Na (31.8–294.5 mg/kg). The content of the remaining minerals in NTQF were within the range reported by the same authors; P (4061.8–4723.9 mg/kg), Cu (3.8–5.9 mg/kg), Mn (18.8–36.6 mg/kg), and Zn (21.3–35.0 mg/kg). The variation may be attributed to differences in genotype and seed maturity [33]. K the most abundant mineral, was 5.09% lower in RTQF, likely due to interactions with other components during heat treatment [25]. Similarly, P content in RTQF was 8.49% lower than in NTQF, possibly due to heat-induced biochemical changes affecting mineral stability [34]. Ahmed et al. [25] reported a 1.59 and 4.34% reduction in K and P content, respectively, in roasted quinoa flour. Similar trends were observed in other roasted seeds, with Tenyang et al. [33] and Babiker et al. [31] reporting reductions of 4.90 and 6.99% in K and P content, respectively, in roasted sunflower and hempseeds. Furthermore, Ca content in RTQF was 8.98% lower than in NTQF, likely due to thermal degradation or Ca binding to heat-induced compounds, reducing its solubility [33]. Mg also decreased by 5.12%, possibly due to structural changes in the matrix during roasting that trap Mg and limit its availability [33,34]. Ahmed et al. [25] reported 2.02 and 5.70% increases in Ca and Mg content, respectively, in roasted quinoa flour. Comparable increases were observed in other roasted grains, with Tenyang et al. [33] reporting 3.19 and 3.63% increases in Ca and Mg in roasted sunflower seeds. These variations are likely due to differences in roasting temperature, duration, quinoa variety, and mineral redistribution during processing, which can either concentrate or degrade minerals [35]. Unlike other minerals, Na content was 4.44% higher in RTQF than in NTQF, likely due to the concentration effect from moisture loss during roasting [33]. Similarly, Ahmed et al. [25] reported an increase of 0.34% in Na content in quinoa after roasting. A similar trend was observed in other roasted grains, with Tenyang et al. [33] and Dhliwayo et al. [36] reporting 5.97 and 15.82% increases in Na content in roasted sunflower seeds and finger millet, respectively. S content showed a minor reduction of 4.57% in RTQF, likely due to interactions with other roasting-induced compounds [33,35]. Similarly, Ahmed et al. [25] reported a slight decrease of 2.54% in S content in quinoa after roasting.

Among the microminerals, Fe experienced the most substantial reduction, decreasing by 45.23% in RTQF, likely due to the formation of insoluble Fe complexes during roasting [1]. Zn content remained relatively stable, with only a 1.26% decrease in RTQF, suggesting its resilience to heat treatment [1]. Mn content was 13.72% lower in RTQF than in NTQF, maybe due to thermal degradation or binding to other heat-induced compounds, reducing its bioavailability [33]. A similar trend was reported by Beniwal et al. [1] where roasting decreased Fe and Zn content in quinoa and amaranth flours by 49.59 and 13.59%, and 28.82 and 21.14%, respectively. Comparable reductions have been observed in roasted quinoa flours, with Ahmed et al. [25] reporting 5.85 and 2.02% lower Fe and Mn content, respectively. Cu content was 2.82% lower in RTQF than in NTQF, indicating minimal losses from thermal processing. Although Al, B, and Mo and are not essential for nutrition, their interactions with different minerals can affect the bioavailability, absorption, and overall physiological balance of these minerals. Al showed the largest reduction of 83.45% in RTQF. This significant decrease is beneficial, as high Al levels can pose health risks. Similarly, B content was 15.11% lower in RTQF compared to NTQF. A similar trend was observed in other roasted flours, with Ahmed et al. [25] and Babiker et al. [31] reporting 2.08 and 4.97% reductions in B content in roasted quinoa and hempseed flours, respectively. Mo levels remained below detectable limits (<0.16 mg/kg) in both flours, indicating that roasting had no measurable impact on its content [37].

#### 3.1.5. Microstructure and Elemental Analysis

**Figure 1:**
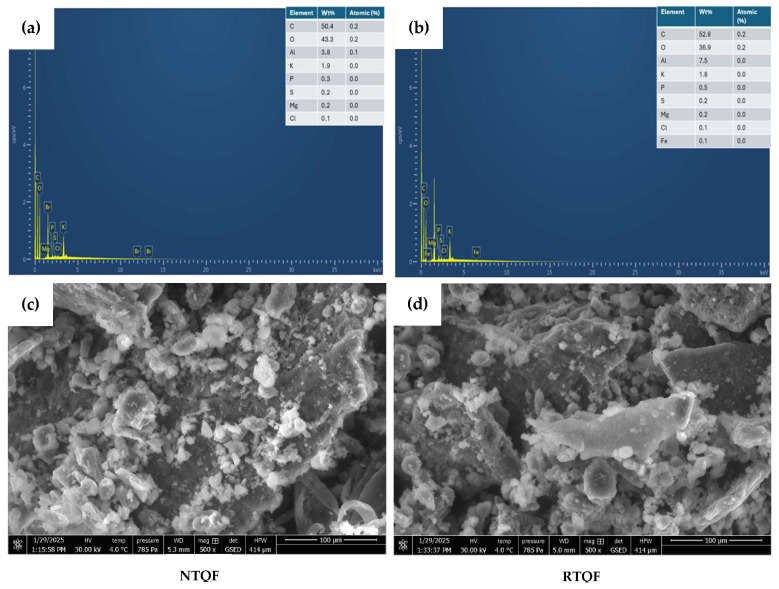
Energy-dispersive X-ray spectra showing the mapping of elemental carbon (C), oxygen (O), potassium (K), phosphorus (P), sulfur (S), chlorine (Cl), iron (Fe), and magnesium (Mg), in (**a**) native tri-color quinoa flour (NTQF) and (**b**) roasted tri-color quinoa flour (RTQF), and scanning electron microscopy (SEM) micrographs (500× magnification) of (**c**) NTQF and (**d**) RTQF.

SEM-EDS examined the surface morphology and elemental composition of NTQF and RTQF. Elemental mapping (Figure 1a,b) identified carbon (C), oxygen (O), Al, K, P, S, Cl, and Mg in both samples, while Fe was detected only in RTQF. The uniform distribution of C suggests a consistent organic composition, while the reduction in O content after roasting indicates moisture loss, which is a common effect of thermal processing [6]. Elemental analysis of RTQF showed a 4.8% increase in C and a 14.8% decrease in O, likely due to partial degradation of carbohydrates and moisture loss. Al content nearly doubled in RTQF, inconsistent with the ICP-OES results. K levels remained relatively stable, with a minor decrease in RTQF of 5.3%. P showed a minor increase, while Mg and Cl remained stable, indicating that these elements were less affected by roasting [38]. Differences between the SEM-EDS and ICP-OES results highlight variations in analytical techniques, where SEM-EDS provides localized surface composition, while ICP-OES measures bulk elemental content, which can be influenced by sample preparation and matrix effects [39].

SEM analysis showed significant morphological variations between NTQF and RTQF (Figure 1c,d. NTQF displayed densely packed particles with fewer visible pores and a compact structure, indicating an intact matrix with minimal physical disruption. Conversely, RTQF showed increased porosity, fragmentation, and a rougher, more irregular surface, suggesting that roasting caused structural breakdown. The formation of cracks and a more open structure in RTQF can be attributed to moisture evaporation, starch granule disintegration, and protein denaturation, which are common effects of thermal processing [2]. These structural modifications align with previous studies on heat-treated grains, where roasting led to increased porosity, reduced particle integrity, and disruption of starch-protein interactions [2,10]. Kheto et al. [2] and Goszkiewicz et al. [40], noted that heat-induced expansion and moisture loss resulted in a looser, more fragmented structure in roasted quinoa, black rice, and sunflower seeds, respectively. The observed differences between NTQF and RTQF indicate that roasting modifies the microstructure of quinoa flour, potentially enhancing its functional properties, such as hydration, solubility, and enzymatic digestibility.

#### 3.1.6. ANFs

The primary ANFs in quinoa are phytic acid and saponins, while tannins, trypsin inhibitors, and oxalates are present in smaller amounts [15]. Roasting reduced all the ANFs studied (Table 1). The phytic acid content in NTQF was within the range (2.9–7.9%) reported by Sharma et al. [7], though it was higher than the 1.30% reported by Marie et al. [8] for raw quinoa flour, and notably lower than the values reported by Manzanilla-Valdez et al. [23] for black (1.97 g/100 g), yellow (2.13 g/100 g) and red (2.21 g/100 g) quinoa. Phytic acid content significantly decreased by 36.05% in RTQF, likely due to their degradation with heat treatment [4]. Similarly, Marie et al. [8] and Sharma et al. [7], respectively, reported 20.37 and 18.0% lower phytic acid content in roasted quinoa flour. A similar trend was reported by Dhliwayo et al. [36], where roasting decreased phytic acid content in roasted finger millet, cowpea, and orange maize by 61.53, 79.06, and 91.17%, respectively. Saponin content in NTQF was notably lower than values reported by Beniwal et al. [1] (1.42 g/100 g) and Manzanilla-Valdez et al. [23] in black, yellow, and red quinoa (83.27, 95.51, and 96.82 mg/g, respectively), while it was considerably higher than that reported by Marie et al. [8] (5.63 mg/100 g) for raw quinoa. These variations are likely attributed to varietal differences, limited pre-processing, such as polishing or washing, which preserves surface saponins [36]. The quinoa used in our study was unpolished. Roasting also led to a nominal and non-statistically significant reduction of 2.05% in saponin content. This minimal change may be due to the relative heat stability of saponins, which resist degradation under standard roasting conditions [8]. Similarly, Marie et al. [8] and Beniwal et al. [1] reported reductions of 9.41 and 54.93%, respectively, in saponin content after roasting quinoa. Comparable reductions have been observed in other roasted grains, with Beniwal et al. [1] reporting a 46.84% reduction in roasted amaranth and Dhliwayo et al. [36] reporting a 61.53% reduction in saponin content in roasted finger millet. These variations may be attributed to differences in roasting temperature, duration, and grain composition, which influence the extent of saponin degradation.

In addition, tannin content of NTQF was higher than the values reported by Beniwal et al. [1] (0.57 mg/100 g), Manzanilla-Valdez et al. [23] (2 mg/100 g), and Marie et al. [8] (36.80 mg/100 g) in white, and black quinoa, respectively. This variation may be attributed to varietal differences, seed coat pigmentation, and extraction efficiency, which influence tannin concentration. Roasting also showed a minor decrease of 7.41% compared to NTQF, although this was not statistically significant. The reduction may be attributed to thermal degradation, oxidation, or polymerization of tannins, which can alter their extractability [1]. Marie et al. [8] and Beniwal et al. [1] observed a 14.48 and 57.89% decrease in tannin content in roasted quinoa and amaranth, respectively. Moreover, oxalate content in NTQF was within the range (3.96–7.15 mg/g) reported by Manzanilla-Valdez et al. [23], in three quinoa varieties. Roasting resulted in a significant reduction of 28.78% in oxalate content, likely due to the thermal degradation or alteration of these compounds [41]. Dhliwayo et al. [36] observed reduced oxalate content in finger millet, cowpea, and orange maize by 31.07, 80.61, and 92.80%, respectively. Furthermore, TIA in NTQF was substantially higher than the range (0.35–0.46 TIU/mg) reported by Manzanilla-Valdez et al. [23] in quinoa, and within the range (0.17–15.09 TIU/mg) reported for raw quinoa by Tavano et al. [42]. Roasting resulted in a slight non-significant reduction of 8.53% in TIA, likely attributed to partial thermal denaturation of trypsin inhibitors. Ahmed et al. [25] observed a 28% reduction in TIA in roasted black quinoa seeds.

#### 3.1.7. Color and Water Activity

The L* value of NTQF was substantially lower than the values reported by Beniwal et al. [1] (76.38) and Kheto et al. [2] (82.86), while the a* and b* values were considerably higher than the values reported by Beniwal et al. [1] (0.62 and 15.28) and Kheto et al. [2] (0.34 and 27.51), likely due to seed pigmentation, as tri-color quinoa contains red and black seeds with higher concentrations of anthocyanins and betalains, which influence color parameters. RTQF was darker, more reddish, and more yellow in color than NTQF. Roasting did not significantly affect the L* value, as evidenced by a mere 0.63% reduction attributed to nominal browning that occurred during roasting (Table 1). A reduced L* value indicates a darker color. Other authors have noted reductions in L* value in roasted quinoa flour of 5.12, and 16.34% by Beniwal et al. [1] and Norouzian et al. [10], respectively. The a* value increased by 6.59% in RTQF compared to NTQF, likely due to Maillard reaction and caramelization generating new pigmented compounds during roasting. Similarly, Marie et al. [8] and Norouzian et al. [10] reported 3.79 and 23.09% increase in a* values in roasted quinoa, respectively. Furthermore, the b* value increased by 3.28% in RTQF, likely due to roasting-induced release of bound phenolics and breakdown of starch structures, both fostering polymerization reactions that form new yellow–brown pigments [43]. A similar trend was observed in b* value, with Marie et al. [8] and Beniwal et al. [1] reporting increases of 3.79 and 23.09%, respectively, in roasted quinoa.

The a_w_ of NTQF was nearly 2- and 3-fold lower than the 0.43 and 0.60 reported by Marie et al. [8], and Norouzian et al. [10], respectively, likely due to milling intensity (particle size) and post-processing drying methods. The a_w_ of RTQF was 26.09% lower than that of NTQF, suggest a reduced amount of “free” or due to dehydration. Lower a_w_ is beneficial food are less susceptible to microbial growth and hydrolytic reactions, thereby enhancing the flour’s shelf stability [4]. Norouzian et al. [10] and Marie et al. [7], respectively, reported 10.0 and 37.21% lower a_w_, in roasted quinoa flour.

#### 3.1.8. Thermal Properties

The Ton, Tp, and Te of NTQF were notably higher than reported by Dong et al. [30] (65.05, 72.78, and 81.09 °C, respectively) and Kheto et al. [2] (99.84, 106.98, and 116.47 °C, respectively), likely attributed to starch composition, and degree of crystallinity, which influence thermal transitions [2] (Table 1). Following roasting, Ton, Tp, and Te of RTQF increased by 5.05, 5.33, and 5.05%, respectively, compared to NTQF. The higher thermal transition temperatures in RTQF can be attributed to structural modifications, such as starch-lipid complex formation, which requires higher heat energy to initiate and complete gelatinization [44]. Dong et al. [30] and Kheto et al. [2], reported increases in Ton, Tp, and Te in roasted quinoa by 50.41, 48.17, and 45.34%, as well as 31.95, 19.16, and 24.79%, respectively. Conversely, ΔH decreased by 39.48% in RTQF, suggesting a lower energy requirement for gelatinization, while Dong et al. [30] noted a 75.75% reduction in roasted quinoa flour. Reduced ΔH may indicate partial thermal degradation of starch or disruption of crystalline structures [30].

#### 3.1.9. Secondary Structure Analysis

**Figure 2:**
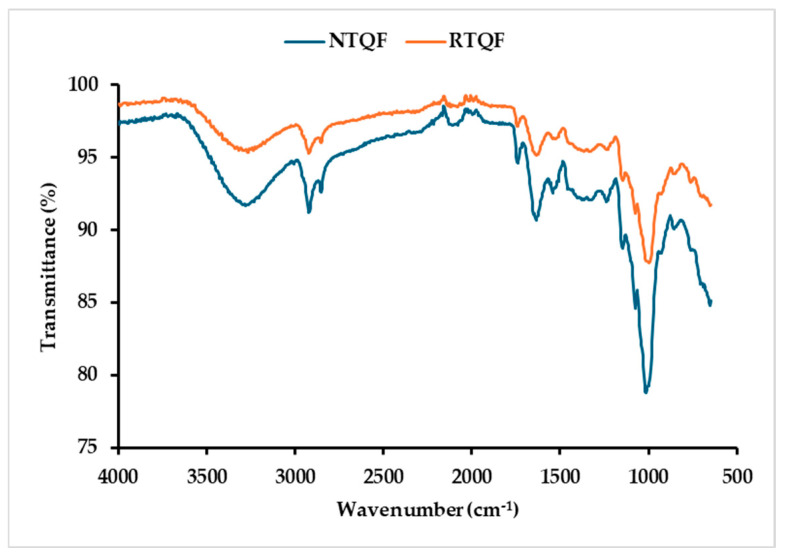
Fourier transform infrared (FTIR) spectra of native tri-color quinoa flour (NTQF) and roasted tri-color quinoa flour (RTQF).

FTIR spectroscopic analysis revealed substantial alterations in RTQF (Figure 2). The FTIR spectrum of NTQF exhibited deeper/intense bands and lower transmittance than RTQF across several functional groups. The higher transmittance (weaker absorption) in the RQTF spectrum reflect moisture loss, protein denaturation, lipid oxidation, and carbohydrate transformation with roasting. Four primary spectral regions displayed notable variations: 3600–3200, 3000–2800, 1745–1540, and 1240–1000 cm^−1^, corresponding to transformations in key functional groups. The broad band at 3309 cm^−1^, attributed to hydroxyl (O-H) stretching, showed higher transmittance in RTQF due to heat-induced moisture loss [2]. Kheto et al. [2] observed similar OH reductions in roasted grains and ascribed it to water evaporation and OH alterations. The 3000–2800 cm^−1^ region, with characteristic peaks at 2981 and 2858 cm^−1^, assigned to methyl (CH_3_) and methylene (-CH_2_) (alkyl) stretching, also less intense in RTQF, suggesting lipid oxidation and partial protein degradation, potentially contributing to flavor development and structural modifications [43]. The region between 1745 and 1540 cm^−1^ included C=O stretching of carbonyl groups (1744 cm^−1^), amide I (1636 cm^−1^), and amide II (1606 cm^−1^) bands. The intense peak in NTQF indicate intact protein secondary structures and stable carbonyl compounds, whereas the decreased in intensity of these peaks in RTQF suggest protein unfolding, denaturation, cross-linking, and carbonyl degradation. These spectral alterations are consistent with Maillard reaction-related transformations during roasting [2]. Furthermore, the 1240–1000 region is a key part of the fingerprint region. The peaks at 1241 and 1017 cm^−1^ are associated with C-O stretching in carbohydrates and amide III vibrations. The less intense peaks in RTQF suggest partial starch degradation, partial gelatinization and formation of Maillard reaction products [2]. Overall, roasting modified the molecular structure of tri-color quinoa flour, and such changes may enhance digestibility while potentially altering techno-functional properties important for food applications [9].

### 3.2. Protein Quality

#### 3.2.1. Amino Acid Composition

Both flours were rich in isoleucine and leucine (Table 3). The content of EAA such as; phenylalanine, methionine, leucine, and lysine in NTQF were considerably higher than the values reported by Shi et al. [5] (0.15–0.16, 0.54–0.61, 0.85–0.96, and 0.79–1.10%, respectively), while valine content was lower than the 4.78% reported by these same authors. This discrepancy is likely due to differences in quinoa varieties, as amino acid profiles can vary significantly among cultivars [45]. Furthermore, the content of non-essential amino acids (NEAA) in NTQF, including glutamic acid, aspartic acid, serine, tyrosine, and alanine was higher than the ranges reported by Shi et al. [5] (2.16–2.50, 1.20–1.50, 0.63–0.78%, 0.37–0.47, and 0.55–0.66%, respectively), while tyrosine content was lower than the 2.74% reported by these same authors. Roasting both significantly and non-significantly affected the content of amino acids (Table 3). It reduced EAA, NEAA, and total amino acids (TAA) content by 6.25, 27.24, and 14.92%, respectively, due to heat-induced degradation and Maillard reaction. It only increased the content of three EAA, including threonine (1.69%), lysine (3.92%), and valine (18.33%), likely due to protein hydrolysis or precursor conversion during roasting [46]. Despite the slight increase in lysine content, and its bioavailability may still be reduced due to Maillard reaction products, which can negatively impact digestibility and bioavailability [45,47]. Tekgül Barut et al. [45] reported a lower increase (7.05%) in valine content in roasted chickpea. Phenylalanine and methionine, on the other hand, showed substantial decreases of 31.68 and 39.10%, respectively. The reduction in phenylalanine was lower, while that of methionine was higher than the 3.93 and 0.96%, respectively reported by Shi et al. [5] in raw quinoa flour. This may negatively impact the EAA and reduce protein quality [47]. Despite the reduction in TAA, the ratio to EAA-to-TAA (E/T) improved by 9.99%, and the Fischer’s ratio increased by 2.10%, indicating that, overall roasting slightly improved the amino acid profile. Furthermore, the NEAA exhibited the most substantial decline (27.24%) compared to EAA, with glutamic acid, aspartic acid, and serine decreasing by 27.09, 37.48, and 53.68, respectively. Tekgül Barut et al. [45] reported reduced aspartic acid, glutamic acid, and serine in roasted chickpea by 8.34, 12.44 and 46.62%, respectively. These reductions may negatively affect the flour’s techno-functional properties, such as solubility and water-holding capacity. However, alanine remained stable, suggesting it resistance to thermal degradation [5,45]. The content of arginine and tyrosine were substantially increased with roasting by 19.28 and 56.12%. Tekgül Barut et al. [45] also reported increased tyrosine content in roasted chickpea, by 27.38%. In addition, the content of sulfur-containing amino acids (SAA), hydrophobic amino acids (BAA), hydrophilic amino acids (PAA), and aromatic amino acids (AAA) were reduced in RTQF.

#### 3.2.2. AA Score, EAAI and p-BV

The requirements for EAA were determined for various age groups of humans (Table 4). With the exception of a few EAA for infants and preschoolers, the flours met or were beyond the requirements for the population. Three protein nutritional indexes of the flours were estimated, including AA score, EAAI and p-BV. AA score is a key measure of protein quality, assessing the level of each EAA in a sample relative to the reference pattern recommended by the FAO. The AA score for NTQF and RTQF across different age groups are also presented in Table 4. Overall, AA scores were lower for infants compared to the other age groups. AA scores ranged from 0.72 to 2.48 in NTQF and 0.74 to 2.46 in RTQF for the populations of interest. Both flours were limiting in lysine for all the populations of interest. Manzanilla-Valdez et al. [23] reported AA score of 0.32–0.45 for adults in three varieties of quinoa using the WHO/FAO/UNU 2007 scoring pattern. Similarly, Li et al. [48] reported AA scores of 0.65–2.0 (infants: 0–6 mon.), 0.5–3.0 (children: 10–12 y), 0.70–2.85 (adults: >18 y) in quinoa flour variants using the Joint FAO/WHO 1971 scoring pattern. Venlet et al. [49] also reported AA scores of 0.98–1.81 in infants (0–6 mon) and 1.00–2.04 in preschool children or children (1–2 y) in quinoa using the WHO/FAO/UNU (2007) scoring pattern for infants (0–6 mon) and preschool children (1–2 y). In their study, valine was identified as the limiting amino acid in both age groups. Using the FAO/WHO 1991 scoring pattern, Stone et al. [9] also reported limiting AA scores for raw green lentil (0.72), chickpea (1.01), navy bean (0.88), and yellow pea (0.85). The authors also reported lower limiting AA score for roasted chickpea (0.97), navy bean (0.79), and yellow pea (0.84) but a slightly higher value for green lentil (0.73). The limiting amino acid in their raw and roasted flours were tryptophan, except for navy bean, which was methionine + cysteine. Kaur & Prasad [50] also reported AA scores for raw and roasted chickpea flour using the WHO/FAO/UNU (2007) scoring pattern in infants (0.58–1.21 and 0.56–1.15) and adults (0.61–1.49 and 0.62–1.37), respectively. The limiting amino acid in both flours were methionine + cysteine across both age groups.

Furthermore, the EAAI measures the overall protein quality based on EAA composition compared to a reference protein. A higher EAAI indicates a more balanced EAA profile and reflects superior nutritional quality. Based on EAAI values, proteins are classified as high-quality (EAAI > 0.90), useful protein sources (EAAI = 0.70–0.80), or of inadequate nutritional quality (EAAI < 0.70) [21]. The EAAI values for NTQF ranged from 0.99 in infants to 1.24 in adults. These values were within the range (0.57–2.41) reported by Manzanilla-Valdez et al. [23] in three quinoa varieties. The EAAI values for RTQF were slightly lower across all age groups, ranging from 0.93 in infants to 1.16 in adults. The lower EAAI in RTQF is likely due of heat-induced amino acid losses, which have been observed in other heat-treated grains [45]. Kaur & Prasad [50] reported a lower EAAI of 0.83 in roasted chickpea, reflecting variability in amino acid stability among different legumes and processing conditions. Moreover, the p-BV, which estimates protein utilization efficiency, ranged from 0.96 to 1.23 in the NTQF and 0.89–1.15 in RTQF (Table 4). These values fall within the range (0.51–2.51) reported by Manzanilla-Valdez et al. [23] in quinoa flours. Kaur & Prasad [50] reported lower values for raw (0.75) and roasted (0.79) chickpea. Similar to the EAAI, NTQF had a higher p-BV across the age groups compared to RTQF. Both flours, despite lower EAAI and p-BV post-roasting, maintained a 0.9 or higher value, indicating high-quality protein sources that are likely to be efficiently absorbed by the human body.

#### 3.2.3. IVPD and IVPDCAAS

The IVPD value of NTQF (Table 3) was within the range (72.9–78.7%) reported for quinoa [5], comparable to that of red (76.90%), yellow (77.61%), and black (77.69%) quinoa flours [23], as well as the range observed in raw pulses (75.01–84.85%) [51]. Roasting had no significant effect on IVPD but slightly increased it by 4.08%, likely due to reduction in ANFs, which interfere with protein digestion. Additionally, heat treatment may have caused partial denaturation, making protein structures more accessible to digestive enzymes [25]. Comparable increases have been observed, with Stone et al. [9] reporting a 2.55, 1.17, and 4.61% increases in roasted chickpea, green lentil, and yellow pea, respectively. Furthermore, RTQF showed significantly higher IVPDCAAS values than NTQF across all age groups, ranging from 0.59 to 0.75%, with increases of 7.27, 8.33, 7.69 and 8.70% for infants, preschool children, adolescents, and adults, respectively (Table 4). This increase is likely due to heat-induced protein denaturation during roasting, which reduces ANFs, thereby enhancing protein digestibility and amino acid availability [25]. A similar trend was reported by Stone et al. [9], in which roasting increased IVPDCAAS in green lentil, navy bean, and yellow pea flours, by 2.52, 2.37, and 3.31%, respectively.

### 3.3. Techno-Functional Properties

#### 3.3.1. Protein Solubility

**Figure 3:**
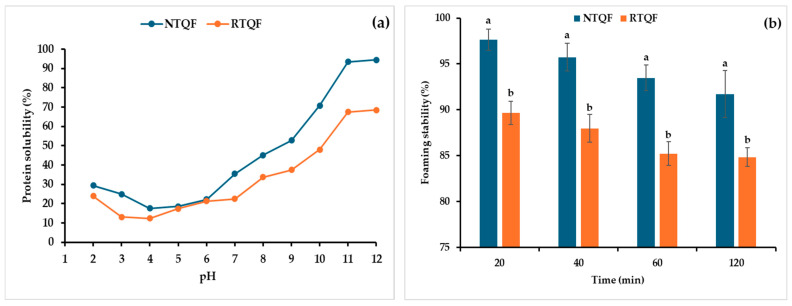
Protein solubility profile (**a**) and foam stability (**b**) of native tri-color quinoa flour (NTQF) and roasted tri-color quinoa flour (RTQF). The foams were generated at pH 7 (at 10 mg/mL).

Protein solubility is an important parameter that affects dispersion in aqueous systems and is essential in emulsification, foaming, and protein digestibility [9]. Protein solubility is influenced by the PAA-BAA balance and the thermodynamics of protein-solvent interactions [52]. As shown in Figure 3a, minimum protein solubility of both NTQF and RTQF occurred at pH 3–6. Solubility was lowest at pH 4, the isoelectric point (*pI*), where protein aggregation and precipitation occur due to no net charge. Higher protein solubility in the alkaline region, with notably higher values observed at high alkalinity, are likely due to higher negative charge repulsion, which promotes stronger protein-water interactions [52]. It should be noted that the flours had other components which can interfere with protein solubility. Roasting reduced protein solubility compared to NTQF across all tested pH levels, notably beyond pH 6. At pH 7, RTQF had 36.56% lower solubility, likely due to protein denaturation, aggregation, and cross-linking caused by heat exposure [52]. In comparison, Beniwal et al. [1], reported even larger reductions in protein solubility of 57.29 and 67.28% for roasted quinoa and amaranth flours at pH 7, respectively. However, Stone et al. [9] reported 42.20, 55.62, and 62.20% increases in roasted green lentil, chickpea, and navy bean flours at pH 7, respectively. These decreases in protein solubility corresponded to changes in secondary structure, suggesting that roasting induces protein alterations. The amide I and II peaks (1500–1700 cm^−1^) exhibited reduced transmittance, pointing to protein denaturation, cross-linking, and structural rearrangement. Such modifications may have contributed to decreased protein solubility by promoting aggregation and reducing protein-water interactions [2].

#### 3.3.2. WAC and OAC

Both WAC and OAC are important techno-functional parameters that influence moisture and fat retention, which are critical for improving texture, mouthfeel, and sensory quality of various food products [1,53]. The WAC of NTQF was notably higher than values reported by Beniwal et al. [1] (1.25%), and Kheto et al. [2] (1.04%), possibly due to varietal differences. Roasting significantly increased WAC by 24.26% (Table 5), likely due to structural modifications in starch and protein, which enhance water-binding sites and improve hydration properties [1]. FTIR analysis supports this finding, showing carbohydrate transformations in the fingerprint region (900–1200 cm^−1^), suggesting partial starch gelatinization, leading to improved water retention [2]. Similar increases in WAC were reported by Sharma et al. [7] (21.57%) and Kheto et al. [2] (22.12%) for roasted quinoa flour. Comparable increases have been observed in other roasted grains, with Byarugaba et al. [54] reporting a 2.63% increase in roasted common beans flour and Stone et al. [9] reporting 18.30, 38.33, and 62.86% increases in green lentil, navy bean, and chickpea flours, respectively. Furthermore, the OAC of NTQF was slightly lower than reported by Sharma et al. [7] (2.49%), but slightly higher than that reported by Beniwal et al. [1] (1.73%) and Kheto et al. [2] (1.09%). This variation may be attributed to differences in lipid content or processing conditions. Roasting also led to a 2.76% increase in the OAC (Table 5), primarily due to structural changes in proteins and lipids, which improve their ability to bind and retain oil [1]. FTIR analysis further supports this, revealing modifications in the 2800–3000 cm^−1^ region, associated with lipid oxidation or degradation, which likely contributed to the increased oil-binding capacity. Similarly, Kheto et al. [2] and Beniwal et al. [1], reported increases of 7.34 and 50.29%, respectively, in roasted quinoa flour. Comparable trends have been observed in other roasted grains, with Byarugaba et al. [54] reporting a 9.64% increase in roasted common bean flour and Stone et al. [9] reporting 4.48, 38.73, and 39.91% higher values in roasted green lentil, chickpea, and navy bean flours, respectively.

#### 3.3.3. EC and ES

EC is another important techno-functional properties. It reflects the ability of certain materials to stabilize oil-water systems by forming and maintaining a cohesive barrier at the interface [7]. The EC of NTQF was substantially higher than values reported by Sharma et al. [7] (44.58%), and Kheto et al. [2] (57.06%), while ES also exceeded the 44.05% reported by Sharma et al. [7]. These variations may be due to inherent structural and compositional differences or variety-specific functional properties. Roasting significantly reduced EC by 47.58% compared to NTQF (Table 5), likely due to heat-induced protein denaturation and structural modifications, which may have impaired the proteins’ ability to stabilize emulsions [6]. Similarly, Kheto et al. [2] reported a 16.05% decrease in EC in roasted quinoa flour. A similar trend was reported by Stone et al. [9], where the EC of roasted yellow pea and navy bean were reduced by 2.27 and 8.44%, respectively. Similarly, ES is another important techno-functional property that measures the ability of an emulsion to resist phase separation over time, reflecting the durability of the protein-stabilized interface [7]. Roasting also led to a 43.58% decrease in ES, likely due to protein aggregation and reduced surface activity, which may have weakened the proteins’ ability to maintain stable emulsions [6]. Kheto et al. [2] reported an 8.53% decrease in the ES of roasted quinoa flour, while Stone et al. [9], reductions of 18.95 and 26.19% in roasted lentil and yellow pea, respectively. The reductions in EC and ES correspond with FTIR findings, which showed decreased amide I and II transmittance (1500–1700 cm^−1^), indicating protein denaturation and cross-linking (Figure 2). The structural changes caused by roasting likely disrupted protein solubility and flexibility, which are critical for emulsion formation [2,43].

#### 3.3.4. FC and FS

FC reflects a material’s ability to generate foam under specific processing conditions, whereas FS describes its effectiveness in maintaining the foam structure over time. The FC of NTQF was considerably higher than the 6.66% reported by Kheto et al. [2]. Stone et al. [9] reported higher FC for green lentil (102.2%), chickpea (200.0%), navy bean (164.4%), and yellow pea (195.6%), compared to our study. FC was significantly reduced by 34.96% in RTQF (Table 5), likely due to heat-induced protein unfolding and aggregation, which impaired the protein’s ability to entrap and stabilize air bubbles [52]. Similarly, Kheto et al. [2] reported a 22.67% decrease in FC in roasted quinoa flour, while Stone et al. [2], noted 7.63, 28.73, and 34.43%, decreased FC in roasted green lentil, yellow pea, and navy bean, respectively. In addition, FS gradually decreased over the 2 h study period in both flours, with RTQF exhibiting greater foam instability. After 40, 60, and 120 min, it decreased by 2.31, 3.67, and 4.90% in RTQF, compared to 1.72, 3.58, and 4.07% in NTQF (Table 5 and Figure 3b). This may be due to limited viscosity of the proteins, along with the slightly lower saponin content, which reduced surface activity and bubble stabilization [50]. The FS of NTQF at 60 min exceeded the 8.30 and 39.30% reported by Anuhya & Dobhal [55] and Ghumman et al. [52], for raw quinoa flour also after 60 min. Ghumman et al. [52] reported a substantial reduction (44.5%) in FS of roasted quinoa flour, similar to the observations by Stone et al. [9] for roasted green lentil (48.33%), chickpea (53.70%), navy bean (65.72%), and yellow pea (81.45%). Furthermore, interference from and interactions between other components apart from protein molecules, can affect both FC and FS.

#### 3.3.5. SC

The SC is a measure of the ability of materials to absorb water and swell. It affects viscosity, thickness, and texture of foods [7]. The SC of NTQF was notably lower than the 10.56% reported by Sharma et al. [7], likely due to a higher proportion of water-absorbing components in their flour compared to this study. Roasting significantly reduced the SC by 17.74% (Table 5), likely due to the thermal degradation of starch granules and denaturation of proteins, which reduces the ability of starches and proteins to absorb water and swell [53]. Similarly, Sharma et al. [7] reported a 7.95% reduction in SC in roasted quinoa flour. Comparable reductions have been observed in other roasted grains, with Nabubuya et al. [53] reporting 9.42% decrease in roasted amaranth seed flours, while Benmeziane-Derradji et al. [6] reported a 4.76% increase in roasted lentil flour.

#### 3.3.6. LGC

LGC provides a measure of the gelling ability of a material, and it is essential in food systems that require structure and thickness [6]. As shown in Table 5, no gel formation was observed in either flour at 2–8% flour concentration, while firm gels developed at ≥14%. While NTQF formed a gel at 10%, RTQF required a minimum concentration of 12% to form a gel. The higher LGC of RTQF may be due to heat-induced protein denaturation and reduced SC, which hinder gel network formation [6]. Despite the higher protein content in RTQF, roasting likely disrupted protein interactions and altered starch functionality, requiring a higher concentration for gel formation. These changes align with FTIR findings, which showed reduced amide I and II transmittance (1500–1700 cm^−1^), indicating protein cross-linking that may have limited protein flexibility and water-binding capacity. Additionally, modifications in the fingerprint region (900–1200 cm^−1^) suggest starch alterations, which may have further weakened gel matrix formation [9]. Unlike quinoa, roasted lentil and faba bean flours exhibited lower LGC values, indicating improved gelation after roasting [56]. Roasted lentil and faba bean flours formed gels at 8 and 12%, respectively, compared to 12 and 16% in their raw flours. This suggests that roasting enhanced protein interactions in legumes, while in quinoa, excessive protein cross-linking and starch modifications hindered gel formation [26].

### 3.4. Interactions

**Figure 4:**
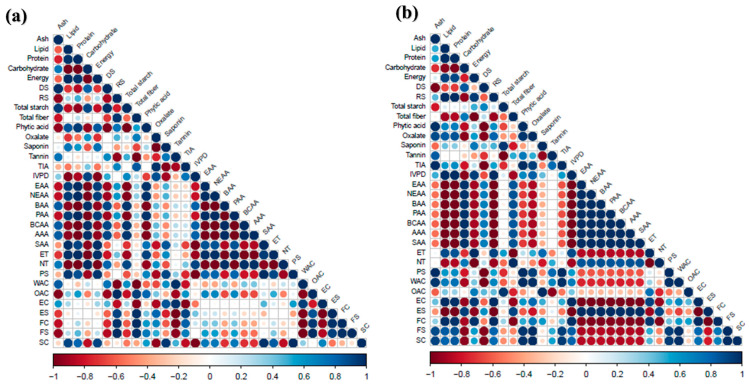
Pearson’s correlation matrix of (**a**) native tri-color quinoa flour (NTQF), and (**b**) roasted tri-color quinoa flour (RTQF) showing interactions between physicochemical, nutritional and techno-functional properties (digestible starch (DS), resistant starch (RS), trypsin inhibitor activity (TIA), in vitro protein digestibility (IVPD), essential amino acids (EAA), non-essential amino acids (NEAA), hydrophobic amino acids (BAA), hydrophilic amino acids (PAA), branched chain amino acids (BCAA), aromatic amino acids (AAA), sulfur-containing amino acids (SAA), ratio of essential amino acids to total amino acids (ET), and ratio of non-essential amino acids to total amino acids (NT), protein solubility (PS), water absorption capacity (WAC), oil absorption capacity (OAC), emulsifying capacity (EC), emulsifying stability (ES), foaming capacity (FC), foaming stability (FS), and swelling capacity (SC).

The relationships between the measured properties of NTQF and RTQF were evaluated using Pearson correlation analysis (Figure 4). The correlation matrices indicated mixed correlations between the parameters. In NTQF, IVPD was negatively correlated with phytic acid (*r* = −0.682) and oxalate (*r* = −0.874), while in RTQF, these correlations were stronger (*r* = −0.996 and −0.827, respectively), as roasting reduced these inhibitors. Additionally, IVPD showed a strong positive correlation with EAA in both NTQF (*r* = 0.944) and RTQF (*r* = 0.997). Carbohydrate content was negatively correlated with IVPD in both NTQF (*r* = −0.952) and RTQF (*r* = −0.953), inferring that starch-protein interactions may hinder protein digestibility. Conversely, lipid content showed a positive correlation with IVPD (*r* = 0.999 in NTQF and RTQF), suggesting that lipids may aid digestion by reducing protein aggregation. Energy content was positively correlated with lipids (*r* = 0.927 in RTQF), as roasting may have concentrated energy-dense components. SAA was strongly correlated with IVPD in RTQF (*r* = 1.000) compared to NTQF, inferring that roasting improved SAA digestibility. EAA and lipid content maintained a strong correlation (*r* = 0.999 in RTQF), as roasting-induced lipid-protein interactions affect amino acid availability. Roasting significantly altered the relationships among quinoa flour’s nutritional, physicochemical, and functional properties. While it enhanced IVPD, reduced ANFs, and improved hydration properties, it also reduced protein solubility, emulsion stability, and foaming properties due to protein denaturation. WAC and OAC were positively correlated in both flours (*r* = 0.995 for NTQF, *r* = 0.988 for RTQF), suggesting that factors that affect water retention also influenced lipid interactions. However, the increased porosity in RTQF likely contributed to its higher WAC and OAC, as structural breakdown allowed better absorption. Protein solubility exhibited positive correlations with EC and FC in NTQF (*r* = 0.553 and 0.554, respectively), but these relationships were weaker in RTQF (r = 0.505 and 0.506, respectively) due to heat-induced protein denaturation. Protein solubility and ES showed a strong negative correlation in both flours (*r* = −0.952 in NTQF, −0.999 in RTQF), indicating that while proteins in the roasted flour can form emulsions, they are less stable over time. FS and FC showed a strong negative correlation in RTQF (*r* = −0.946) compared to NTQF, suggesting that roasting decreased the ability of proteins to maintain stable foams. EC and ES were strongly negatively correlated in RTQF (*r* = −0.999), reinforcing that while roasting improved emulsification, it compromised stability.

## 4. Conclusions

This study presented the impact of dry roasting on the physicochemical, nutritional, and techno-functional properties of tri-color quinoa. Roasting enhanced the lipid, protein, carbohydrate, energy, starch, and saturated fatty acid contents of tri-color quinoa while reducing the content of most minerals, anti-nutritional factors, and several amino acids. Roasting also improved protein digestibility and in vitro protein digestibility-corrected AA score. AA score, EAAI and p-BV were calculated for tri-color quinoa for the first time. Among the techno-functional properties studied, roasting enhanced water and oil absorption capacity but reduced protein solubility, emulsification, and foaming properties, likely due to protein denaturation. Despite these limitations, the results suggest strong potential for roasted tri-color quinoa flour as a promising ingredient in gluten-free and plant-based formulations. Future studies will focus on incorporating the flours in bakery products, protein-enriched snacks, and other plant-based alternatives.

## Figures and Tables

**Table 1:** Physico-chemical and thermal properties of tri-color quinoa flours.

Parameter	NTQF	RTQF
**Physico-Chemical**		
Moisture (%)	7.99 ± 0.10 ^a^	4.97 ± 0.05 ^b^
Ash (%)	2.45 ± 0.08 ^a^	2.54 ± 0.05 ^a^
Lipid (%)	5.59 ± 0.03 ^a^	5.80 ± 0.15 ^a^
Protein (%)	14.97 ± 0.25 ^a^	15.10 ± 0.10 ^a^
Carbohydrate (%)	69.01 ± 0.30 ^b^	71.59 ± 0.25 ^a^
Energy (kcal/100 g)	386.22 ± 0.06 ^b^	398.97 ± 0.81^a^
Digestible starch (%)	5.36 ± 0.52 ^a^	5.48 ± 0.16 ^a^
Resistant starch (%)	1.48 ± 0.14 ^a^	1.63 ± 0.13 ^a^
Total starch (%)	6.84 ± 0.40 ^a^	7.11 ± 0.13 ^a^
Total dietary fiber (%)	17.31 ± 0.88 ^a^	15.04 ± 0.04 ^b^
Phytic acid (mg/g)	2.94 ± 0.10 ^a^	1.88 ± 0.13 ^b^
Oxalate (mg/g)	5.42 ± 0.33 ^a^	3.86 ± 0.04 ^b^
Saponin (mg/g)	13.64 ± 0.77 ^a^	13.36 ± 1.0 ^a^
Tannin (mg/g)	1.35 ± 0.08 ^a^	1.25 ± 0.02 ^a^
TIA (TIU/mg)	2.93 ± 0.72 ^a^	2.68 ± 0.57 ^a^
Water activity	0.23 ± 0.00 ^a^	0.17 ± 0.01 ^b^
L*	68.20 ± 0.26 ^a^	67.77 ± 0.15 ^a^
a*	7.44 ± 0.21 ^b^	7.93 ± 0.15 ^a^
b*	31.37 ± 0.12 ^b^	32.40 ± 0.35 ^a^
ΔE*	-	1.26 ± 0.22
**Minerals (mg/kg)**		
Ca	544.50 ± 0.14 ^a^	495.60 ± 0.14 ^b^
K	10,800.50 ± 0.71 ^a^	10,250.50 ± 0.71 ^b^
Mg	1993.50 ± 0.71 ^a^	1891.50 ± 0.71 ^b^
Na	13.07 ± 0.02 ^b^	13.65 ± 0.01 ^a^
P	4087 ± 0.54 ^a^	3740 ± 0.85 ^b^
S	1783 ± 0.35 ^a^	1701.50 ± 0.71 ^b^
Cu	5.68 ± 0.01 ^a^	5.52 ± 0.01 ^b^
Fe	110.25 ± 0.35 ^a^	60.38 ± 0.01 ^b^
Mn	26.31 ± 0.04 ^a^	22.70 ± 0.01 ^b^
Zn	26.26 ± 0.01 ^a^	25.93 ± 0.01 ^b^
Mo	<0.16	<0.16
Al	69.61 ± 0.02 ^a^	11.52 ± 0.01 ^b^
B	10.72 ± 0.01 ^a^	9.10 ± 0.01 ^b^
**Thermal Properties**		
T_on_ (°C)	172.05 ± 1.84 ^b^	180.73 ± 2.37 ^a^
Tp (°C)	172.19 ± 1.36 ^b^	181.36 ± 2.66 ^a^
T_e_ (°C)	175.87 ± 0.83 ^b^	184.76 ± 2.94 ^a^
ΔH (J/g)	152.54 ± 12.30 ^a^	92.32 ± 8.72 ^b^

Trypsin inhibitor (TIA), lightness (L*), red/green (a*), and yellow/blue (b*) coordinates, and total color difference (ΔE*), onset temperature (To), peak temperature (Tp), end temperature (Te), and enthalpy of gelatinization (ΔH) of native tri-color quinoa flour (NTQF) and roasted tri-color quinoa flour (RTQF). Values are means ± SD. Different superscript letters in the same row indicate significant (*p* < 0.05) differences.

**Table 2:** Fatty acid composition of tri-color quinoa flours.

Fatty Acids (%)	NTQF	RTQF
Myristic acid (C14:0)	0.15 ± 0.03 ^b^	0.21 ± 0.01 ^a^
Palmitic acid (C16:0)	8.15 ± 0.63 ^b^	9.93 ± 0.16 ^a^
Stearic acid (C18:0)	3.10 ± 0.40 ^b^	4.04 ± 0.02 ^a^
Oleic acid (C18:1)	40.30 ± 5.19 ^b^	54.19 ± 1.09 ^a^
Linoleic acid (C18:2n-6)	26.18 ± 2.42 ^a^	22.81 ± 0.55 ^a^
Arachidic acid (C20:0)	0.50 ± 0.04 ^a^	0.49 ± 0.01 ^a^
Linolenic acid (C18:3n-3)	3.25 ± 0.46 ^a^	2.29 ± 0.17 ^b^
Docosanoic acid (C22:0)	0.88 ± 0.05 ^a^	0.92 ± 0.02 ^a^
Erucic acid (C22:1ω9)	0.38 ± 0.09 ^a^	0.18 ± 0.02 ^b^
Arachidonic (C20:4n-6)	0.05 ± 0.03 ^a^	0.02 ± 0.01 ^b^
SFA	16.11 ± 1.19 ^a^	17.03 ± 0.04 ^a^
MUFA	44.29 ± 4.40 ^b^	56.74 ± 1.15 ^a^
PUFA	39.60 ± 4.99 ^a^	26.23 ± 1.18 ^b^

Saturated fatty acids (SFA), monounsaturated fatty acids (MUFA), and polyunsaturated fatty acids (PUFA) in native tri-color quinoa flour (NTQF) and roasted tri-color quinoa flour (RTQF). Values are means ± SD. Different superscript letters in the same row indicate significant (*p* < 0.05) differences.

**Table 3:** Amino acid composition and protein digestibility of tri-color quinoa flours.

Amino Acid (g/100 g)	NTQF	RTQF
Essential Amino Acids (EAA)		
Histidine (His)	1.66 ± 0.03 ^a^	1.55 ± 0.02 ^b^
Isoleucine (IIe)	7.45 ± 0.35 ^a^	7.39 ± 0.04 ^a^
Leucine (Leu)	6.78 ± 0.10 ^a^	6.24 ± 0.08 ^b^
Lysine (Lys)	4.08 ± 0.11 ^a^	4.24 ± 0.13 ^a^
Methionine (Met)	3.35 ± 0.01 ^a^	2.04 ± 0.04 ^a^
Phenylalanine (Phe)	2.62 ± 0.03 ^a^	1.79 ± 0.01 ^b^
Threonine (Thr)	2.37 ± 0.04 ^a^	2.41 ± 0.02 ^a^
Tryptophan (Trp)	0.71 ± 0.03 ^a^	0.66 ± 0.00 ^b^
Valine (Val)	3.60 ± 0.08 ^b^	4.26 ± 0.05 ^a^
**Non-Essential Amino Acids (NEAA)**		
Alanine (Ala)	1.05 ± 0.06 ^a^	1.05 ± 0.16 ^a^
Arginine (Arg)	1.66 ± 0.53 ^a^	1.98 ± 0.52 ^a^
Aspartic acid (Asx)	5.07 ± 0.26 ^a^	3.17 ± 0.46 ^b^
Cysteine (Cys)	0.18 ± 0.00 ^a^	0.20 ± 0.00 ^a^
Glutamic acid (Glx)	2.51 ± 0.20 ^a^	1.83 ± 0.00 ^b^
Glycine (Gly)	1.31 ± 0.11 ^a^	1.12 ± 0.24 ^a^
Proline (Pro)	1.57 ± 0.01 ^a^	1.38 ± 0.01 ^b^
Serine (Ser)	8.16 ± 0.48 ^a^	3.78 ± 0.03 ^b^
Tyrosine (Tyr)	1.39 ± 0.02 ^b^	2.17 ± 0.03 ^a^
EAA	32.63 ± 0.49 ^a^	30.59 ± 0.31 ^b^
NEAA	22.91 ± 0.65 ^a^	16.67 ± 0.46 ^b^
Total amino acids (TAA)	55.55 ± 0.15 ^a^	47.26 ± 0.77 ^b^
E/T ratio (%)	58.75 ± 0.01 ^b^	64.72 ± 0.00 ^a^
Aromatic amino acids (AAA)	4.73 ± 0.02 ^a^	4.63 ± 0.04 ^a^
Branched chain amino acids (BCAA)	17.84 ± 0.52 ^a^	17.90 ± 0.09 ^a^
Fischer’s ratio	3.77 ± 0.10 ^b^	3.87 ± 0.02 ^a^
Hydrophobic amino acids (BAA)	26.88 ± 0.36 ^a^	25.93 ± 0.55 ^b^
Hydrophilic amino acids (PAA)	27.15 ± 0.52 ^b^	24.81 ± 0.31 ^a^
Sulfur-containing amino acids (SAA)	3.54 ± 0.01 ^a^	2.24 ± 0.04 ^b^
IVPD	76.30 ± 2.84 ^a^	79.41 ± 1.81 ^a^

Essential amino acids (His, Ile, Leu, Lys, Met, Phe, Thr, Trp, and Val), non-essential amino acids (Asx, Glx, Ser, Gly, Ala, Arg, Tyr, Cys, and Pro), E/T (ratio of essential amino acids to total amino acids), aromatic amino acids (Phe, Trp and Tyr), branched chain amino acids (Ile, Leu and Val), Fischer’s ratio (ratio of BCAA to AAA), hydrophobic amino acids (Ile, Leu, Met, Phe, Trp, Val, Ala and Pro), hydrophilic amino acids (His, Lys, Thr, Arg, Asp, Cys + Glu + Ser, and Tyr), sulfur-containing amino acids (Cys and Met), and in vitro protein digestibility (IVPD) of native tri-color quinoa flour (NTQF) and roasted tri-color quinoa flour (RTQF). Values are means ± SD. Different superscript letters in the same row indicate significant (*p* < 0.05) differences.

**Table 4:** Amino acid requirements and scores for various age groups for tri-color quinoa flours.

Amino Acid (mg/g Protein)	Reference AA Profile ^1^				NTQF	RTQF
	0.5 y (Infants)	1–2 y (Preschoolers)	11–14 y (Adolescents)	>18 y (Adults)		
**His**	20	18	16	15	16.56 ± 0.30 ^a^	15.48 ± 0.15 ^b^
**Ile**	32	31	30	30	74.52 ± 3.47 ^a^	73.93 ± 0.36 ^a^
**Leu**	66	63	60	59	67.81 ± 0.97 ^a^	62.44 ± 0.77 ^b^
**Lys**	57	52	48	45	40.85 ± 1.06 ^a^	42.45 ± 1.33 ^a^
**SAA**	28	26	23	22	35.36 ± 0.06 ^a^	22.37 ± 0.44 ^b^
**AAA**	52	46	41	38	40.14 ± 0.44 ^a^	39.65 ± 0.40 ^a^
**Thr**	31	27	25	23	23.67 ± 0.40 ^a^	24.06 ± 0.17 ^a^
**Trp**	8.5	7.4	6.5	6.0	7.13 ± 0.28 ^a^	6.63 ± 0.01 ^b^
**Val**	43	42	40	39	36.04 ± 0.81 ^b^	42.60 ± 0.47 ^a^
**Animo Acid Score**						
**His**	-	-	-	-	0.83	0.77
**Ile**	-	-	-	-	2.33	2.31
**Leu**	-	-	-	-	1.03	0.95
**Lys**	-	-	-	-	0.72	0.74
**Met + Cys**	-	-	-	-	1.31	0.83
**Phe + Tyr**	-	-	-	-	0.77	0.76
**Thr**	-	-	-	-	0.76	0.78
**Trp**	-	-	-	-	0.84	0.78
**Val**	-	-	-	-	0.84	0.99
**EAAI (I** **nfants)**	-	-	-	-	0.99 ± 0.01 ^a^	0.93 ± 0.01 ^b^
**p-BV (Infants)**	-	-	-	-	0.96 ± 0.01 ^a^	0.89 ± 0.01 ^b^
**IVPDCAAS (Infants)**	-	-	-	-	0.55 ± 0.01 ^b^	0.59 ± 0.02 ^a^
**His**	-	-	-	-	0.92	0.86
**Ile**	-	-	-	-	2.40	2.38
**Leu**	-	-	-	-	1.08	0.99
**Lys**	-	-	-	-	0.79	0.82
**Met + Cys**	-	-	-	-	1.41	0.89
**Phe + Tyr**	-	-	-	-	0.87	0.86
**Thr**	-	-	-	-	0.88	0.89
**Trp**	-	-	-	-	1.02	0.95
**Val**	-	-	-	-	0.88	1.04
**EAAI (Preschool children)**	-	-	-	-	1.08 ± 0.01 ^a^	1.01 ± 0.01 ^a^
**p-BV (Preschool children)**	-	-	-	-	1.06 ± 0.01 ^a^	0.99 ± 0.01 ^b^
**IVPDCAAS (Preschool children)**	-	-	-	-	0.60 ± 0.02 ^a^	0.65 ± 0.02 ^a^
**His**	-	-	-	-	1.03	0.97
**Ile**	-	-	-	-	2.48	2.46
**Leu**	-	-	-	-	1.13	1.04
**Lys**	-	-	-	-	0.85	0.88
**Met + Cys**	-	-	-	-	1.54	0.97
**Phe + Tyr**	-	-	-	-	0.98	0.97
**Thr**	-	-	-	-	0.95	0.96
**Trp**	-	-	-	-	1.10	1.02
**Met + Cys**	-	-	-	-	0.90	1.07
**EAAI (** **Adolescents)**	-	-	-	-	1.17 ± 0.01 ^a^	1.10 ± 0.01 ^b^
**p-BV (** **Adolescents)**	-	-	-	-	1.16 ± 0.01 ^a^	1.08 ± 0.01 ^b^
**IVPDCAAS (** **Adolescents)**	-	-	-	-	0.65 ± 0.02 ^b^	0.70 ± 0.02 ^a^
**His**	-	-	-	-	1.10	1.03
**Ile**	-	-	-	-	2.48	2.46
**Leu**	-	-	-	-	1.15	1.06
**Lys**	-	-	-	-	0.91	0.94
**Met + Cys**	-	-	-	-	1.61	1.02
**Phe + Tyr**	-	-	-	-	1.06	1.04
**Thr**	-	-	-	-	1.03	1.05
**Trp**	-	-	-	-	1.19	1.11
**Val**	-	-	-	-	0.92	1.09
**EAAI (A** **dults)**	-	-	-	-	1.24 ± 0.01 ^a^	1.16 ± 0.01 ^b^
**p-BV (** **Adults)**	-	-	-	-	1.23 ± 0.01 ^a^	1.15 ± 0.01 ^b^
**IVPDCAAS (** **Adults)**	-	-	-	-	0.69 ± 0.02 ^b^	0.75 ± 0.02 ^a^

Histidine (His), isoleucine (Ile), leucine (Leu), lysine (Lys), sulfur-containing amino acids (SAA: Met + Cys), aromatic amino acids (AAA), threonine (Thr), tryptophan (Trp), valine (Val) of native tri-color quinoa flour (NTQF) and roasted tri-color quinoa flour (RTQF). ^1^ Scoring pattern (mg/g protein requirement) of infants, preschool children, adolescents, and adults (males and females combined) adapted from Food and Agriculture Organization [22]. The amino acid score (AA score) was calculated as the ratio of the amino acid content to their reference values; essential amino acid index (EAAI) was calculated as $\sqrt[{9}]{Product\;of\;AA\;score\;of\;all\;the\;EAA}$; in vitro protein digestibility-corrected amino acid score (IVPDCAAS) was calculated as the product of the limiting amino acid (lysine) and IVPD, and predicted biological value (p-BV) was calculated as 1.08 × AAI − 11.70. Means in the same with different superscripts are significantly (*p* < 0.05) different.

**Table 5:** Techno-functional properties of tri-color quinoa flours.

Parameter	NTQF	RTQF
WAC (g/g)	2.02 ± 0.08 ^b^	2.51 ± 0.13 ^a^
OAC (g/g)	1.81 ± 0.02 ^b^	1.86 ± 0.02 ^a^
EC (%)	84.77 ± 0.95 ^a^	44.44 ± 3.38 ^b^
ES (%)	59.89 ± 0.94 ^a^	33.79 ± 3.51 ^b^
FC (%)	37.67 ± 2.52 ^a^	24.50 ± 1.80 ^b^
SC (%)	3.72 ± 0.21 ^a^	3.06 ± 0.11 ^b^
**LGC—Flour Concentration (%, *w*/*v*)**		
2%	-	-
4%	-	-
6%	-	-
8%	-	-
10%	+	-
12%	+	+
14%	++	+
16%	+++	++
18%	+++	+++
20%	+++	+++

Water absorption capacity (WAC), oil absorption capacity (OAC), emulsifying capacity (EC), emulsifying stability (ES), foam capacity (FC), foam stability (FS), swelling capacity (SC) of native tri-color quinoa flour (NTQF), and roasted tri-color quinoa flour (RTQF). No gel (-), gel (+), firm gel (++), very firm gel (+++). Values are means ± SD. Different superscript letters in the same row indicate significant (*p* < 0.05) differences.

## Data Availability

The raw data supporting the conclusions of this article will be made available by the authors on request.
